# A Pilot Study of an Innovative Hand Exoskeleton: Nuada Glove

**DOI:** 10.7759/cureus.47411

**Published:** 2023-10-21

**Authors:** Rui Santos, Rodrigo Correia, Yuriy Mazin, Hugo Andrade, Filipe Quinaz

**Affiliations:** 1 Physical Medicine and Rehabilitation, Centro de Reabilitação do Norte, Centro Hospitalar Vila Nova de Gaia/Espinho, Vila Nova de Gaia, PRT; 2 Biomedical Engineering and Software Engineering, Faculdade de Engenharia da Universidade do Porto, Vila Nova de Gaia, PRT; 3 Computer Science and Engineering, Universidade da Beira Interior, Covilhã, PRT

**Keywords:** hand disability, activities of daily living, exoskeleton, rehabilitation, assistive technology

## Abstract

The human hand is a crucial anatomical structure, and its complexity enables humans to grasp tools essential for daily life activities. Consequently, hand disabilities have a negative and profound impact on people's autonomy in performing everyday tasks. The Nuada Glove (Nuada, Braga, Portugal) is a wearable exoskeleton robotic device designed to assist individuals with hand impairments or weakness in regaining hand function, particularly in grasping movements. This pilot study aims to evaluate its impact on improving hand function in first-time users with hand disabilities and to assess its usability. A total of 63 participants with hand disabilities were asked to complete a series of exercises that simulated various human grasping motions, initially without any assistance and then with the Nuada Glove. Of the participants, 98% exhibited measurable improvement in hand function in at least one exercise. This pilot study, conducted in a controlled environment, clearly demonstrated the benefits of the Nuada Glove and its ease of use. Future studies should expand from this one to assess the use of the Nuada Glove in a real-world environment with continuous use.

## Introduction

Hand disabilities can significantly impact a person's ability to perform daily activities. The human hand is a complex and versatile tool that allows us to engage in a wide range of tasks, such as grasping objects, writing, typing, using tools, and performing fine motor movements.

Our hands are amazing but are prone to suffer from problems such as weakness, pain, and tremors. These problems can arise due to a wide range of conditions and causes. Because of the scale of the problem, it is difficult to provide an exact global prevalence. Dahaghin and colleagues found a prevalence of hand pain and hand-related disabilities of 13.6% (7.2% men; 17.8% women) in an open population aged 55 years and older [1].

Treatment options may include medications, physical therapy, occupational therapy, splints or braces, assistive devices, surgical interventions, or a combination of these approaches. Exoskeleton gloves have emerged as a promising technology in the field of physical medicine and rehabilitation. As stated by Jacob Rosen et al. [2], who conducted an overview of modern active exoskeletons, hand exoskeletons are frequently complex devices due to the intricate anatomy of the hand (very small size and many degrees of freedom) and the human-robot interaction that they require. They require high levels of multidisciplinary expertise in the fields of electrical engineering, mechanical engineering, computer science, medicine, and anatomy.

Even though these exoskeleton devices target the same anatomical part, i.e., the hand, their design, requirements, and functionality highly differ. These decisions are tightly linked to the intended application. Several studies [2-4] have categorized hand exoskeletons into assistive devices (aimed at replacing impaired hand functioning), rehabilitation systems (aimed at recovering functionality lost due to injury or medical conditions), augmentation exoskeletons (aimed at enhancing the users’ ability above their normal levels), and haptic devices (aimed at monitoring, actuation, or virtual reality).

The Nuada Glove (Nuada, Braga, Portugal) is a wearable exoskeleton robotic device designed to assist individuals with hand impairments or weakness. This device is manufactured under legal requirements and regulated at the European Union Member State level. The main innovation of this device is the actuation method, which enables the combination of the lightweight and ease of use of passive exoskeletons, and the power and adaptability of active exoskeletons. Active exoskeletons require heavy packs of motors to exert force on the anatomical structures of the user, thus their usefulness is limited to specific actions. On the other hand, passive exoskeletons are static devices with very specific functions, but they lack adaptability and provide limited gained function. The Nuada Glove is a hybrid approach. It consists of a mechanical system that locks each finger individually in the grasping position when patients hold objects of any size and shape. First, users encircle the objects either with or without assistance, and the mechanical system impedes them from opening their fingers, effectively enabling them to hold any object with their hands regardless of weakness or other impairment.

Acceptability is a crucial step when integrating a new technology into clinical practice in rehabilitation [5]. Patients’ desire and adoption of soft robotic devices are greatly impacted by how they facilitate the performance of activities of daily living (ADL) and instrumental activities of daily living (iADL) [6,7]. Furthermore, this innovation has the potential to enhance patients' involvement in intensive rehabilitation programs [8]. However, despite all the positive effects, patients value the ease of use the most, as reported by Quin Boser et al. [9]. In their exploratory interview study, the general consensus among participants was on criteria related to grasp patterns, grip strength, wear time, and acceptable bulk/weight, which are characteristics mostly related to ease of use and are precisely the advantages that the Nuada Glove claims to have over other solutions.

Therefore, the aim of the present study was to evaluate the efficiency of Nuada as a single-user hand-function medical device. In addition, with this study, we contribute to solving the issues of the “disconnect between current clinical practice and exoskeleton research findings” as reported by Philip Tran et al. [10].

## Materials and methods

**Figure 1:**
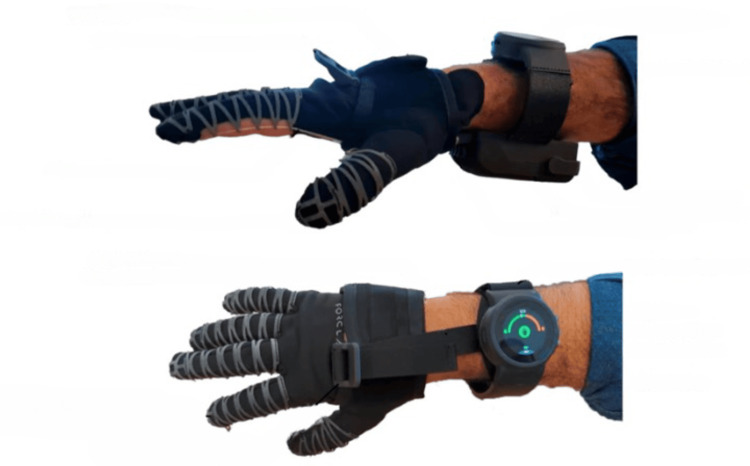
Side and top view of the Nuada Glove.

The protocol's objective is to assess not only the primary grasping function but also various non-functional attributes that impact the potential and acceptance of the Nuada Glove, illustrated in Figure 1. The trial took the form of a case series, involving a series of activities designed to closely simulate ADL. Participants in the study were first-time users of the Nuada Glove and received a 15-minute instructional session on the system.

**Figure 2:**
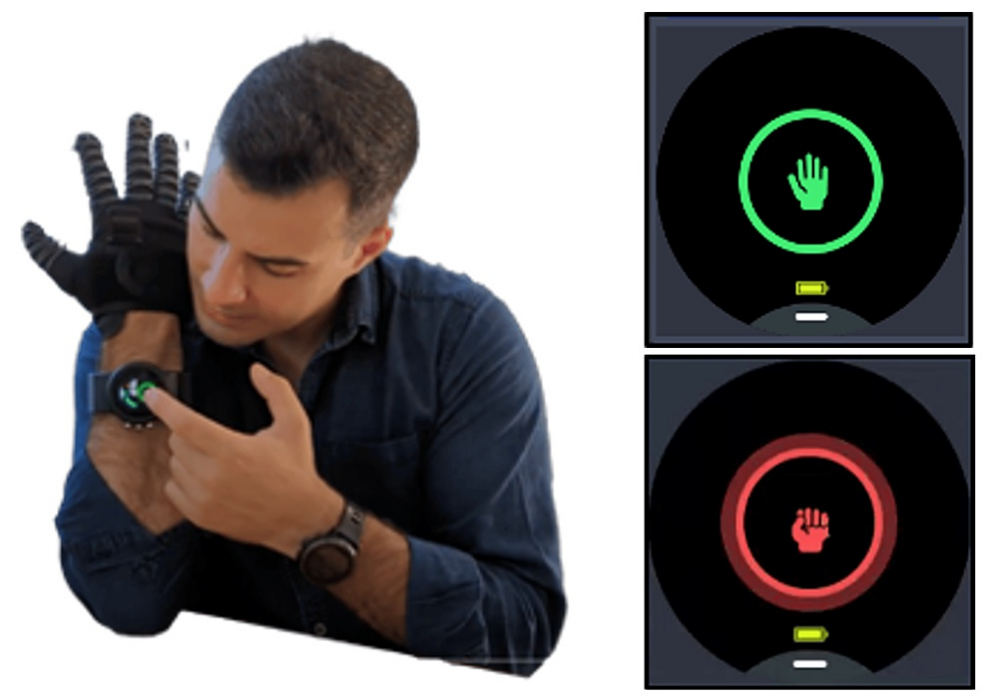
Toggling locking states of the Nuada Glove. Clicking the touchscreen (left) toggles the states (right) between inactive (up) and active (down).

The Nuada Glove is an electromechanical system that consists of several key components: a textile component, mechanical finger structures, artificial tendons, a mechanical system (located under the forearm), and electronics. The glove's textile component features openings in the fingers and palm, specifically designed to allow individuals with severe hand limitations to easily don the Nuada Glove. The interplay between the mechanical finger structures, artificial tendons, and the mechanical system is pivotal to the Nuada Glove's locking mechanism. When inactive, the Nuada Glove functions like a regular glove, enabling users to move their fingers freely. Upon activation, as users close their fingers, the artificial tendons move into the mechanical system but do not come out, enabling the locking mechanism and permitting users to securely grasp objects without requiring muscular effort from their hands. Since the mechanism does not apply any additional force, there is no risk of inadvertently crushing objects. The electronics serve the primary role of controlling the Nuada Glove's locking state, and it boasts remarkable energy efficiency, capable of sustaining a full day of continuous usage. As shown in Figure 2, switching between the locking states of the Nuada Glove is achieved by a simple tap on the touchscreen of the associated smartwatch.

There exist additional advanced features that were intentionally left unexplained to users to avoid overcomplicating their first experience with the Nuada Glove. These features include the ability to increase grasping force with wrist movements and control the locking state through gesture recognition, rather than manual touchscreen input. These advanced features significantly enhance the user experience but do not directly contribute to the study's metrics, which primarily focus on hand function improvement.

For this feasibility and acceptability study, no formal sample size calculation was undertaken. The sample did not statistically represent any population; the participants were people who met the inclusion criteria and were willing to participate in the study.

After a 15-minute explanation of the system's functioning, each participant was asked to perform the exercises twice: in control conditions (no glove) and in intervention conditions (wearing the Nuada Glove), with an exercise time limit of 15 minutes. The exercises were evaluated quantitatively (e.g., time of execution, number of repetitions) and subjectively (e.g., smoothness of movements, effort perceived). The data were recorded by the trial personnel.

The activities aimed to assess two types of grasps: power and precision grasps. The participants were asked to perform each movement five times. These activities evaluated the grasping function of the glove and the activation and deactivation of the Nuada Glove. The movements are listed in Table 1.

**Table 1:** Grasping movements asked in this study.

Power grasps			Precision grasps		
Cylindrical	Spherical	Hook	Pinch	Tripod	Lumbrical
With a cup-like cylinder placed on a table	With a ~5 cm diameter ball placed on a table	With a 5 kg bag	With an object mimicking remote keys	With a pen/pencil, and a sheet of paper	With a card

The evaluation protocol is detailed further in Annex 1. The research team collected data on trial-specific case report forms (CRFs), as shown in Annex 2.

Patients admitted from May 2022 to March 2023 in the North Rehabilitation Center and the São Pedro Continuum Care and Rehabilitation Unit (SPCCRU) were screened and enrolled. They were selected on the basis of the inclusion and exclusion criteria outlined in Table 2.

**Table 2:** Inclusion and exclusion criteria used in this study. AROM: active range of motion; PROM: passive range of motion.

Inclusion criteria	Exclusion criteria
Age > 18 years	Cognitive impairment (a score of 23 or less in the Mini-Mental State Examination)
Acceptance of informed consent	PROM limitations of the upper limb and AROM limitation of shoulder or elbow >50%
Suffer a medical condition with an impact on hand function	Uncontrolled pain

Improvement performance analysis

We define "improvement" as when a patient achieves a higher score by executing an exercise (or set of exercises) with the Nuada Glove compared with the execution of the same tasks without the glove.

It should be noted that the scoring method (ranging from one to five) is not linear, nor does it reflect a proportional improvement. In fact, as long as the patient scores at least 1 score higher in a task when using the Nuada Glove, it already demonstrates the benefit of Nuada. However, improving the score from 1 to 2 holds greater significance and has the most impact on the patient's hand function. In the CRFs used in this study (in Annex 2), a score of 1 generally means "cannot do the task at all," while scoring 2 or higher means "is able to do the task but for a short period of time." If the patient can complete the task, the main function of the Nuada Glove is successfully accomplished. The duration for which the patient can hold the object then depends on other anatomical structures, such as arm or shoulder strength, which are independent of the Nuada Glove. Improved scores above 2 are associated with time, and the amount of extra time is irrelevant to the Nuada Glove, as the system can hold for as long as required. In this study, achieving a maximum score might not be possible due to limitations in other anatomical structures of the arm (shoulder, elbow, and forearm).

Users who reached the maximum score within a certain exercise without the glove and maintained it with the glove were not considered in the "improvement" calculations. This means that the current trial could not measure improvements beyond the maximum score.

All statistical analyses were conducted using the Statistical Package for the Social Sciences (SPSS®) version 23.0 (IBM Corp., Armonk, NY). Only descriptive statistics were performed in this study; therefore no comparisons were made between the groups.

## Results

A total of 63 patients participated in this study, with a mean age of 56 years old. The minimum age was 24 years, and the maximum age was 84 years. Table 3 shows the distribution of the samples by age group.

**Table 3:** Characterization of the data by age group.

Age group (in years)	Percentage
<40	23.8% (n = 15)
40-65	36.5% (n = 23)
>65	39.7% (n = 25)

The patients were evenly distributed by sex, and exhibited similar age characteristics, as illustrated in Tables 4, 5.

**Table 4:** Characterization of the data by sex.

Sex	Percentage	Mean age	% Young adults
Male	50.8% (n = 32)	57	34.4% (n = 11)
Female	49.2% (n = 31)	56	12.9% (n = 4)

**Table 5:** Characterization by pathology.

Pathology	% of participants
Stroke	31.7% (n = 20)
Multiple sclerosis	7.9% (n = 5)
Brachial plexus injury	3.2% (n = 2)
Spinal cord injury	34.9% (n = 22)
Parkinson’s disease	11.1% (n = 7)
Cerebral palsy	3.2% (n = 2)
Guillain-Barré syndrome	7.9% (n = 5)

Regarding user selection, 10 participants were excluded from further processing. Although three users met the inclusion criteria related to decreased hand function, the degree of disability was not noticeable in the exercises associated with this clinical study; they achieved maximum performance without the Nuada Glove, which means that there was no room for improvement with the Nuada Glove. To ensure that this information was not relevant, these users still used the Nuada Glove and maintained the maximum scores, making it impossible to assess the impact of the system on their hand function using the predetermined metrics. In addition, seven patients, who had maximum hand function limitations (i.e., without the glove, they scored the minimum score in all exercises) and maintained the minimum score in all exercises when wearing the glove, were also excluded from further processing. Six of these patients did not have enough arm movement to execute the tasks, both with and without Nuada; thus, because this device acts only on the hand and not on the arm, there was no way to further assess Nuada's impact. The remaining patient did not understand the tasks, both with and without the system.

Improvement performance analysis results

Table 6 displays the percentage of users who demonstrated an increase in hand function, further categorized by hand problem classification.

**Table 6:** Percentage of patients who could use the glove autonomously and had improvements in hand function in any of the exercises, by pathology.

Pathology	% of patients with improvements
Stroke	100% (n = 17)
Multiple sclerosis	100% (n = 5)
Brachial plexus injury	100% (n = 2)
Spinal cord injury	100% (n = 16)
Parkinson’s disease	100% (n = 6)
Cerebral palsy	50% (n = 2)
Guillain-Barré syndrome	100% (n = 5)
Any	98% (n = 54)

From the data, there was only one instance in which a single user (patient number 11), during a specific task (exercise number 1), exhibited worse results with the glove compared with performing the task without any support. The reason for this was that the user suffered from edema, making it impossible to properly wear the glove. This was due to glove size incompatibility and pain, which prevented us from donning (putting on) the system. The patient also had severe limitations in his hand function, which without the glove allowed only a brief ability to perform exercise number 1, but not any of the others.

Despite the overall improvement in hand function, the Nuada Glove may offer better assistance for certain grasping movements. Therefore, the improvement in hand function was also evaluated on the basis of the type of exercise. Table 7 displays the percentage of all patients who improved in each exercise by at least one point. Users who were already at the maximum score and maintained it were excluded from the overall improvement calculation.

**Table 7:** Percentage of patients who had improvements by type of exercise.

Exercise	% of patients with improvements
Grasp cylinder	81% (n = 42)
Grasp sphere	78% (n = 41)
Grasp a bag	92% (n = 43)
Click on button	63% (n = 16)
Grab a pen	71% (n = 41)
Grab a sheet of paper	58% (n = 19)

As observed, Nuada’s added value is not consistent across the measured exercises. This provides us with a clear understanding of the tasks that are easier to perform with Nuada and offers insights into the relationship between the time the user's familiarity with the system and their ability to use specific system features.

## Discussion

The human hand is a crucial tool for engaging in a wide range of tasks, and its complexity enables humans to grasp tools with different shapes and forms. Therefore, hand disabilities greatly impact people's autonomy in daily activities. While various treatments may be employed, few are as promising and have as positive an impact as robotic devices. The Nuada Glove is a wearable exoskeleton robotic device designed to assist individuals with hand impairments or weakness; however, its benefits must be clinically evident to confidently adopt it for widespread use.

The primary objective of this study was to assess the impact of the Nuada system on hand function in first-time users, after a 15-minute explanation of the system's functioning followed by a 15-minute exercise time limit.

The Nuada Glove demonstrated a significant improvement in hand function, with 98% of all users showing quantitative improvement in at least one of the trial exercises. The sole selected participant who did not benefit from the device suffered from hand edema, which limited their ability to wear the glove, particularly when putting it on, and, consequently, to perform exercises with the Nuada Glove. Characteristics such as hand size compatibility with glove size and shape must be considered in future inclusion and exclusion criteria.

The usability and ease of use of this novel device were noteworthy. With a brief explanation and demonstration of the system, the participants were able to successfully execute several exercises involving different types of grasps, which they were unable to perform without the Nuada Glove. A more comprehensive understanding of the system, including its advanced features such as increasing finger closing force based on wrist movement, could further enhance user performance. Interestingly, the lack of knowledge of these advanced features may be the reason for the limited improvement in the task of grabbing a sheet of paper. Therefore, a key factor in the successful use of Nuada may be associated with a deeper knowledge of the system's capacities. Because the Nuada Glove is a mechanical system capable of maintaining hold of objects up to 40 kg, users were expected to hold all objects used in this study. Nevertheless, the efficacy of this device was evident, as most participants demonstrated improvement in hand function.

Several takeaways are relevant for future studies. First, it is important to refine patient selection criteria to make them more objective and reduce the inclusion of patients who cannot benefit from the system. Second, gathering information on why patients did not achieve better results with the glove would be valuable. Verbal communication with the clinical team revealed issues such as a lack of friction between the glove and the cylinder, which was apparently very slippery, and underutilization of Nuada features that could have improved user performance. Finally, while the study was conducted in a controlled environment and closely monitored by medical professionals, no accidents occurred, and no risks were identified, suggesting that the system can be safely used in real-world scenarios involving grasping similar to those objects in the trial.

Regarding insights for future clinical studies, several points should be considered. First, information regarding user product knowledge is crucial. While we limited the education time to 15 minutes, the duration should be adjusted for each user until they feel comfortable and capable of performing simple tasks independently before proceeding to the actual pilot exercises. The total amount of time required should then be measured. Second, providing external verbal clues from the clinical team during the testing of the system in the requested exercises could be beneficial to ensure optimal system use within the available time. Third, although this study did not find a significant relationship between pathologies and exercise results, this may be due to the normalizing effect of the inclusion and exclusion criteria. It would be relevant to conduct pathology-specific trials using different and more tailored user selection processes. In addition, the scoring method could be improved to show a more linear relationship between the impact of the system, its added value, and the scoring value. Finally, because some of the pathologies involve a small sample pool, further studies should focus on those specific diagnostic groups. It is important to ensure that the lack of a detected significant difference between the exercises that Nuada assists with and the hand problem diagnosis is either genuine or imperceptible due to small data sizes.

## Conclusions

The human hand is a crucial structure for engaging in a wide range of tasks, and its complexity enables humans to grasp tools with different shapes and forms. Therefore, hand disabilities significantly impact people's autonomy in daily activities. Various treatments may be employed, but few are as promising and have such a positive impact as robotic devices. The Nuada Glove is a wearable exoskeleton robotic device designed to assist individuals with hand impairments or weakness, but its clinical benefits must be clearly demonstrated to confidently support its widespread adoption.

This pilot study aimed to assess the impact of the Nuada Glove on the improvement of hand function in first-time users with hand disabilities. Participants were asked to complete a series of exercises that mimic different human grasps, initially without any assistance and then with the Nuada Glove. Notably, 98% of users demonstrated quantitative improvement in hand function in at least one exercise. Specifically, the grasping exercises, which are crucial for most daily activities, showed the most significant improvement. The Nuada Glove clearly offers substantial benefits in regaining hand function. This pilot study was conducted in a controlled setting. No incidents and no risks to the patients were identified. This is especially noteworthy considering the brief user education that was provided before the actual use of the product. To further assess the adoption and frequency of use of the Nuada Glove in daily life, it is recommended to expand this pilot to a more continuous and extended trial conducted in a real-world environment.

## Figures and Tables

**Figure 3:**
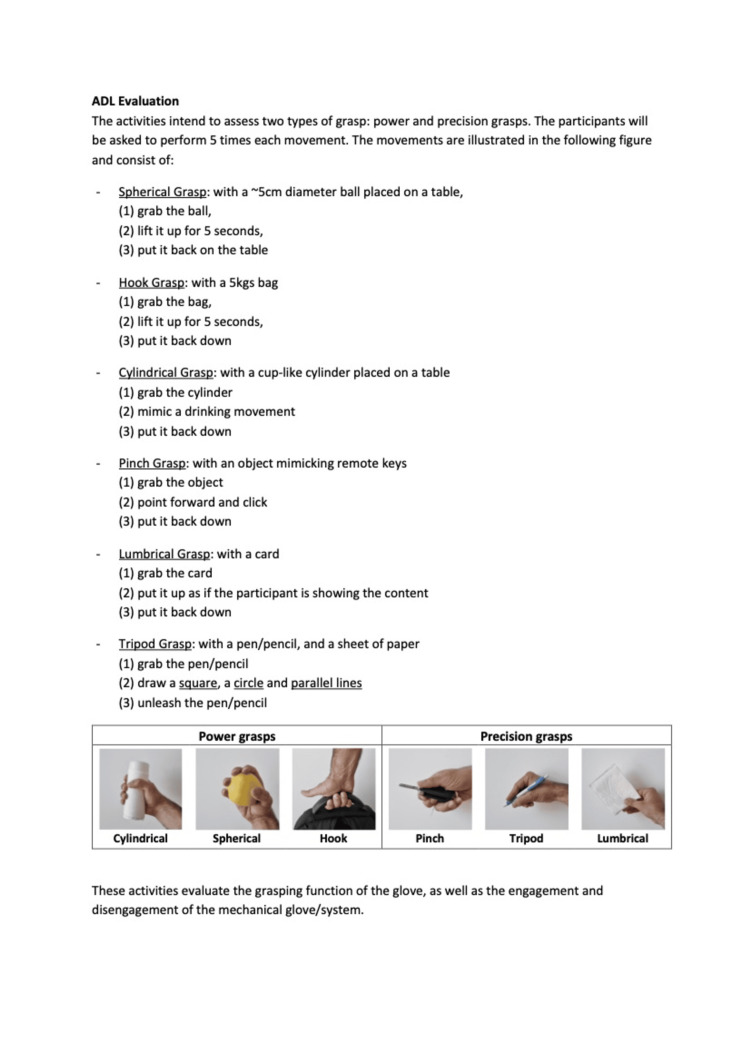
Protocol for the Nuada Glove.

**Figure 4:**
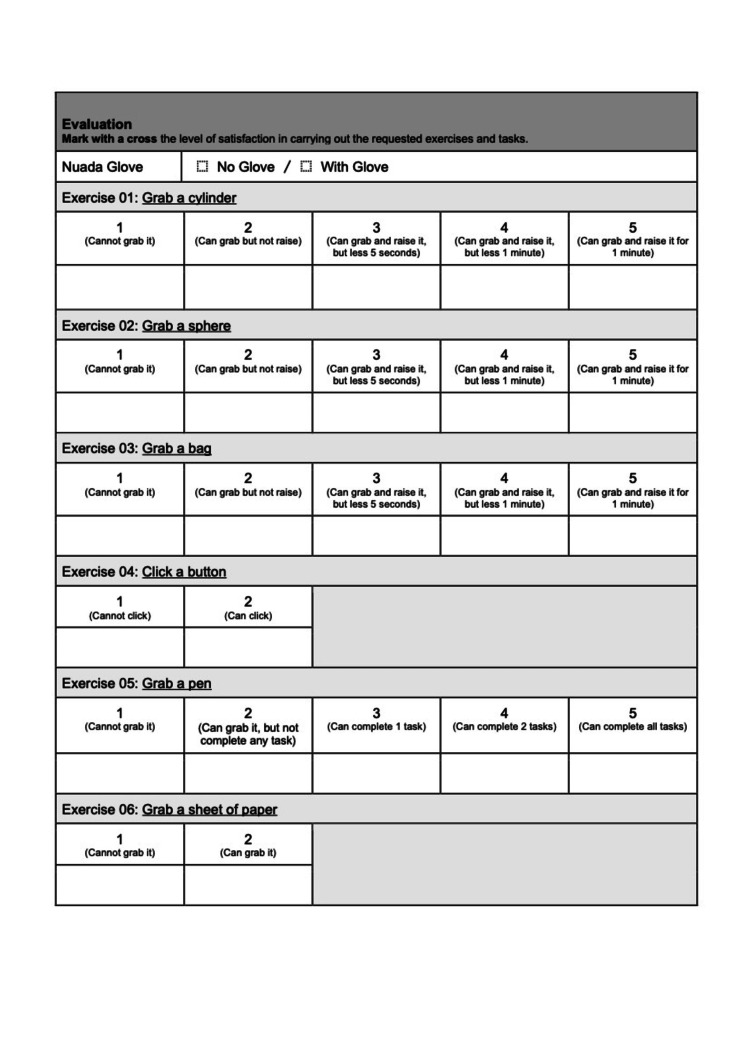
Clinical report form.

## Appendices

Annex 1: evaluation protocol

Annex 2: case report form
